# Quasi-Solid-State Lithium-Sulfur Batteries Assembled by Composite Polymer Electrolyte and Nitrogen Doped Porous Carbon Fiber Composite Cathode

**DOI:** 10.3390/nano12152614

**Published:** 2022-07-29

**Authors:** Xinghua Liang, Yu Zhang, Yujuan Ning, Dongxue Huang, Linxiao Lan, Siying Li

**Affiliations:** Guangxi Key Laboratory of Automobile Components and Vehicle Technology, Guangxi University of Science and Technology, Liuzhou 545006, China; lxh18589873093@163.com (X.L.); yuzhang5332@163.com (Y.Z.); yujuan996@163.com (Y.N.); hdx877348318@163.com (D.H.)

**Keywords:** solid-state lithium-sulfur battery, composite polymer electrolytes, porous carbon

## Abstract

Solid-state lithium sulfur batteries are becoming a breakthrough technology for energy storage systems due to their low cost of sulfur, high energy density and high level of safety. However, its commercial application has been limited by the poor ionic conductivity and sulfur shuttle effect. In this paper, a nitrogen-doped porous carbon fiber (NPCNF) active material was prepared by template method as a sulfur-host of the positive sulfur electrode. The morphology was nano fiber-like and enabled high sulfur content (62.9 wt%). A solid electrolyte membrane (PVDF/LiClO_4_/LATP) containing polyvinylidene fluoride (PVDF) and lithium aluminum titanium phosphate (Li_1_._3_Al_0_._3_Ti_1_._7_(PO_4_)_3_) was prepared by pouring and the thermosetting method. The ionic conductivity of PVDF/LiClO4/LATP was 8.07 × 10^−5^ S cm^−1^ at 25 °C. The assembled battery showed good electrochemical performance. At 25 °C and 0.5 C, the first discharge specific capacity was 620.52 mAh g^−1^. After 500 cycles, the capacity decay rate of each cycle was only 0.139%. The synergistic effect between the composite solid electrolyte and the nitrogen-doped porous carbon fiber composite sulfur anode studied in this paper may reveal new approaches for improving the cycling performance of a solid-state lithium-sulfur battery.

## 1. Introduction

With the increasing energy demand for energy storage equipment in the current market, traditional lithium-ion batteries cannot meet the requirements due to low energy density, poor safety and high cost [1,2]. Therefore, it is imperative to develop and research a new battery system with high specific energy and high safety. The theoretical specific capacity and theoretical specific energy of lithium-sulfur battery can reach 1675 mAh g^−1^, 2600 wh kg^−1^. What’s more, sulfur has obvious advantages in environmental protection, acquisition cost, and so on. It is considered to be the most promising next-generation new energy storage system [3,4]. However, the commercialization of lithium-sulfur batteries still faces some problems [5,6]. Firstly, liquid electrolytes have the safety problem of inflammability and the possibility of explosion, and the lithium dendrite grown from the negative electrode pierces the diaphragm, leading to short circuit [7,8]. Secondly, sulfur has poor electrical conductivity and low utilization rate of active materials. The “shuttle effect” caused by polysulfide dissolution leads to low capacity and coulomb efficiency [1,9].

In order to solve the above problems, more and more attention has been paid to solid-state lithium-sulfur batteries [10,11]. On the one hand, the cathode side requires a high conductivity material to improve contact with low conductivity S. Carbon materials have high electrical conductivity, high specific surface area and excellent mechanical properties, which can provide a conductive network for sulfur and discharge products (Li_2_S) and improve the electrochemical performance of sulfur cathodes [12]. In addition, the introduction of nitrogen doping cannot only significantly improve the electrical conductivity of carbon materials, but also introduce active sites on the surface of carbon materials [13]. Therefore, nitrogen-doped carbon materials can be used in lithium-sulfur batteries as active materials with high electronic conductivity and strong physical and chemical adsorption. On the other hand, there is no solid electrolyte that can meet all the requirements, such as high ionic conductivity at room temperature, a wide electrochemical stability window, good mechanical properties, etc. [14,15,16,17]. The advantages and disadvantages of different solid-state electrolytes are integrated by using composite electrolytes, which provides a new idea for further study of solid-state lithium-sulfur batteries. Polyoxyethylene (PEO) and polyvinylidene fluoride (PVDF) are common polymer electrolyte substrates [18,19,20]. Currently, polyoxyethylene (PEO) has been widely studied in lithium-sulfur batteries, but its ionic conductivity is low at room temperature, and it can only show good ionic conductivity in the amorphous state of 60~90 °C [21]. Compared with PEO, the PVDF electrolyte has better mechanical strength and a higher melting point. Adding inorganic filler to a polymer electrolyte to form CPEs can effectively improve ionic conductivity and lithium-ion transference number. Common inorganic electrolytes include NASICON type, Li_10_GeP_2_S_12_(LGPS) type, Li_x_PON type, Li_2_S-P_2_S_5_ type and Li_7_La_3_Zr_2_O_12_ (LLZO) type [22,23,24,25]. Lithium titanium aluminum phosphate (LATP) is a glass ceramic material with NASICON type three-dimensional network structure, which has the advantages of high mechanical strength, high ionic conductivity, high temperature stability and stability to air and water [26,27]. However, the application of LATP as an electrolyte in batteries is limited by its large interfacial impedance and side effects. By adding a certain amount of nano-scale ceramic materials into the polymer electrolyte, the composite polymer-ceramic electrolyte (CPEs) formed has lower interfacial resistance and higher ionic conductivity [28,29]. It can inhibit the formation of lithium dendrite and the shuttle effect of polysulfide and can be effectively applied to solid-state lithium-sulfur batteries.

In this paper, a nitrogen-doped porous carbon fiber active material (NPCNF) with a microporous structure and nanofiber shape was prepared via template method. The NPCNF/S electrode exhibits excellent performance due to the better electrical conductivity and strong physical and chemical adsorption of carbon and nitrogen doped materials. A PVDF/LiClO_4_/LATP composite solid electrolyte (CPEs) was prepared, which combined the advantages of inorganic electrolytes and polymer electrolytes. It has the characteristics of a wide electrochemical window, high ionic conductivity and stable mechanical properties at room temperature. The assembled quasi-solid lithium sulfur battery was tested at 25 °C and had excellent performance. This study proves that the long cycle performance of a solid-state lithium-sulfur battery is improved at a large magnification rate, which provides ideas for subsequent research.

## 2. Materials and Methods

### 2.1. Materials

The raw materials included PEO-PPO-PEO (P123) (99%, Aladdin, Shanghai, China), C_8_H_2_OO_4_Si (99%, Aladdin, Shanghai, China), HCl (98%, Aladdin, Shanghai, China), C_2_H_4_N_4_ (99%, Aladdin, Shanghai, China), HF (40%, Aladdin, Shanghai, China), S (99%, Aladdin, Shanghai, China), polyvinylidene fluoride (PVDF) (Mw = 600,000, Macklin, Shanghai, China), lithium bisimide (LiClO_4_) (99.99% purity, Aladdin, Shanghai, China), Li_1_._3_Al_0_._3_Ti_1_._7_(PO_4_)_3_ (99.99% purity, Macklin, Shanghai, China).

### 2.2. Preparation of the NPCNF/S Composite

We dissolved 1 g PEO-PPO-PEO (P123) in 6 mL C_8_H_2_OO_4_Si under magnetic stirring. Then, we added 32 mL ethanol and 0.583 mL concentrated hydrochloric acid (HCl, 37%) to the solution. After adding 4 mL of deionized water, wefully stirred the hydrolysis for 2 h. Adding 2.8 g dicyandiamide (DCDA) as carbon source and nitrogen source, the semi-solid colloid was obtained by stirring and drying at 80 °C. The powder was dried overnight at 80 °C to obtain a white powder; then, we calcined N_2_ in a tubular furnace at 1000 °C for 60 min at a heating rate of 3 °C min^−1^. After cooling to room temperature, the sintered powder was poured into 5%HF solution to clean the template. Fully cleaned samples were dried at 60 °C for 12 h to obtain the final product NPCNF. The NPCNF was mixed with elemental S at a mass ratio of 1:2 and calcined at 155 °C for 12 h in a tube furnace under a nitrogen atmosphere. Cooling to room temperature to obtain NPCNF/S.

### 2.3. Preparation of the Composite Solid Electrolyte Membrane (CPEs)

PVDF, LATP and LiClO_4_ powders were vacuum-dried at 60 °C for 24 h. PVDF, LATP, LiClO_4_ and DMF were weighed at the mass ratio of 10:1:0.124:80. PVDF was dissolved in 40 mL DMF and stirred at 55 °C for 1 h to form a transparent viscous solution. LATP and LiClO_4_ were added and stirred for 5~6 h. Finally, the mixed solution was cast into a polytetrafluoroethylene mold and vacuum dried at 60 °C for 24~72 h to obtain flexible electrolyte films with ceramic/polymer composites

### 2.4. Battery Assembly

NPCNF/S, conducting carbon and polyvinylidene fluoride (PVDF), were dissolved in N-methylpyrrolidone (NMP) at a mass ratio of 7:2:1 and stirred to a obtain uniform slurry. The slurry was coated on aluminum foil and dried in a vacuum drying oven for 12 h. The composite electrolyte was cut into discs with a diameter of 18 mm. The 2025-coin cells were assembled and tested. We then added two drops of electrolyte. The electrolyte was 1.0 mol LiTFSI in DOL:DME = 1:1 vol% with 1.0 wt% LiNO_3_.

### 2.5. Characterization

Via X-ray diffraction (XRD, D8-Advance, Bruker, Germany), the material phase was analyzed by measuring the diffraction data in the range of 10~90°. Via thermogravimetric analysis (TGA) measurement in air atmosphere temperature under the condition of 10 °Cmin^−1^, we performed an analysis of material quality, along with the change of temperature. The cathode material was tested via Raman spectroscopy (XploRA PLUS, HORIBA, France) under a 523 nm Raman microscope. The microscopic morphology of the sample was characterized via scanning electron microscope (SEM, Sigma04-55, ZEISS, Germany). The composition and valence of solid electrolyte elements were determined by X-ray photoelectron spectroscopy (XPS, K-alpha, Thermo, America) at 5 kV.

### 2.6. Electrochemical Measurements

The timing current of lithium symmetric battery was tested at a voltage of 0.5 mV, lasting 4000 s, and the formula was as follows:

$$t_{Li+}=Is(\Delta V-I_{0}R_{0})/I_{0}(\Delta V-IsRs)$$

The lithium-ion transfer rate (*t_Li+_*) can be obtained. *I*_0_ and *Is* are current values after DC polarization starts and stabilizes, *R*_0_ and *Rs* are the impedance values before and after the DC polarization, and Δ*V* is the value of the voltage applied to both ends of the battery.

For the ionic conductivity test, battery assembly used SS as a symmetrical battery and electrochemical impedance test together to calculate the ionic conductivity. The frequency range of impedance test is 0.1~106 Hz.

σ=L/S×R

where σ represents the ionic conductivity, L is the thickness of electrolyte, *S* represents the contact area between electrolyte and test electrode (SS) and *R* is the impedance value of battery electrolyte measured by EIS. The battery test system (CT-400, Neware, Hong Kong, China) performed constant current charge–discharge cycle tests between 1.5 and 3 V. At 25 °C, the electrochemical workstation (DH-7000, Donghua, Shanghai, China) was used for cyclic voltammetry (CV) test at 1.5~3 V and 0.2 mV s^−1^. Linear sweep voltammetry (LSV) was used to perform electrochemical window tests at 2~6 V at a scanning rate of 0.1 mV s^−1^.

## 3. Results

**Figure 1:**
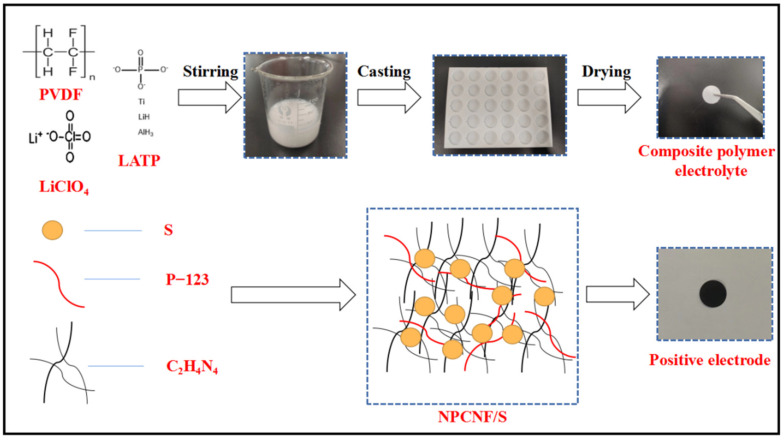
A schematic illustration of the fabrication of the positive electrolyte and CPEs.

Figure 1 shows the manufacturing process of CPEs and NPCNF/S positive poles. The NPCNF material was prepared via the etching template method. Its unique hole structure increased the specific surface area of the material, and it could load more elemental sulfur. After mixing with S, the positive electrode sheet was obtained after the slurry coating. The polymer, lithium salt and inorganic electrolyte were fully dissolved in the mixed solution, and the nanoscale LATP was uniformly combined with PVDF to obtain CPEs.

**Figure 2:**
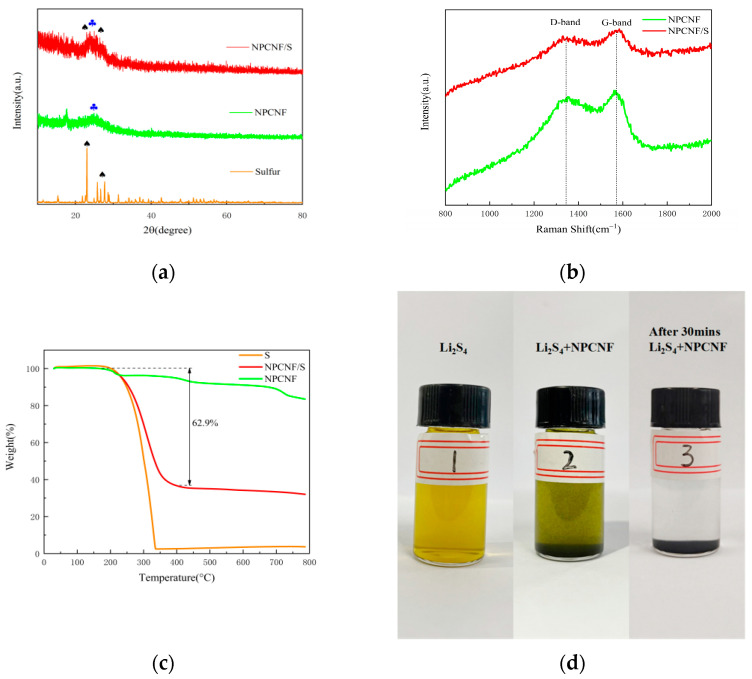
(**a**) XRD tests of the NPCNF and NPCNF/S (**b**) Raman spectra of NPCNF and NPCNF/S (**c**) TGA curves of S, NPCNF and NPCNF/S (**d**) The photograph of the static adsorption test.

**Figure 3:**
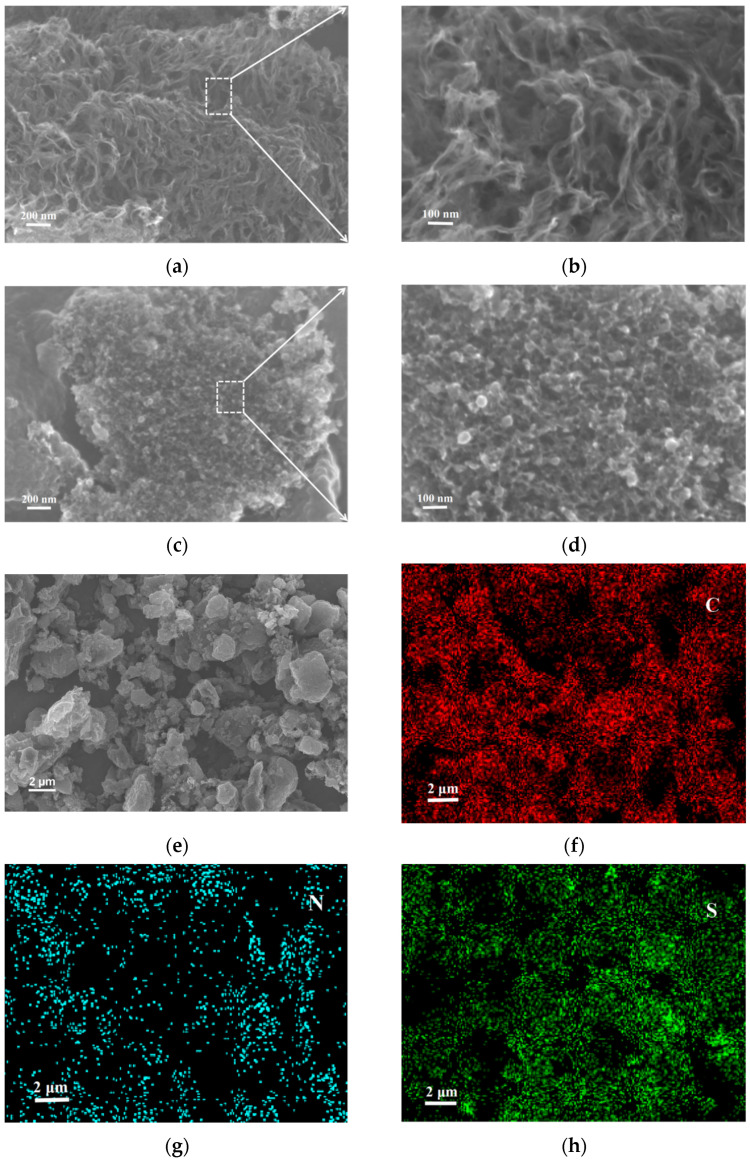
(**a**,**b**) SEM images of NPCNF (**c**,**d**) SEM images of NPCNF/S (**e**) SEM image of NPCNF/S and corresponding elemental mapping images (**f**) C element (**g**) N element (**h**) S element.

XRD patterns of NPCNF and NPCNF/S are shown in Figure 2a. NPCNF/S has an obvious diffraction peak corresponding to elemental S at 23.04°. There is a diffraction peak at 24.8° of NPCNF corresponding to the (002) plane of graphite carbon, which proves that a certain amount of graphite amorphous carbon is formed in the material. The diffraction peak at two places indicates that the characteristics of elemental S and NPCNF are retained in NPCNF/S. NPCNF/S, compared with the diffraction peak of sulfur, was reduced greatly, and this is due to the large amounts of S fully penetrated into the microporous structure of the carbon fiber material [30]. The corresponding morphology can be observed in the SEM figure (Figure 3c,d). Raman spectroscopy was used to test NPCNF and NPCNF/S, as shown in Figure 2b. The D band and G band intensity ratios of NPCNF and NPCNF/S are 1.04 and 1.03, respectively. The differences were small, indicating that the introduction of sulfur particles did not change the graphitization degree of NPCNF. The I_D/G_ values are all greater than 1, indicating that the active material has a high degree of graphite carbonization and good conductivity [31]. In order to determine the sulfur content of the NPCNF/S sample, TGA measurement was carried out, as shown in Figure 2c. The mass change of the sample was measured when the temperature was raised to 800 °C at a heating rate of 10 °C min^−1^ in a nitrogen flow. It can be seen that there was about a 17 wt% amount of weight loss when NPCNF rose to 800 °C, and the elemental sulfur rapidly sublimated to complete disappearance at 250~350 °C. The sulfur content of the NPCNF/S sample is about 62.9 wt%. The sulfur loading and content of the cathode is 0.38 mg cm^−2^. An experiment on adsorption of polysulfide lithium was carried out using NPCNF, as shown in Figure 2d. Firstly, we added Li_2_S_4_ to form a light-yellow solution (Bottle No. 1); 10 mg of NPCNF was added as the No. 2 solution. After standing for 30 min, a clear and transparent liquid was formed in bottle No.3. The pore structure of the NPCNF material had an obvious adsorption and anchoring effect on Li_2_S_4_, which results in an inhibiting “shuttle effect” of lithium-sulfur batteries.

SEM characterization tests were conducted for NPCNF and NPCNF/S, as shown in Figure 3. Figure 3a,b shows that the surface of NPCNF presents an irregular reticular structure resembling nanofiber. After being fully etched by the HF solution, an EDS test analysis of NPCNF shows that no Si element was found in the material, and nano-SiO_2_ particles generated by tetraethyl orthosilicate hydrolysis were cleaned and removed. The holes leftover increase the specific surface area of the material, which is conducive to the load of S and sulfide in the positive electrode. At the same time, polysulfide can be adsorbed through physical action to provide channels for ion transfer in the battery. After loading S, the sample changes from a nanofiber to porous mesoporous structure, but the original carbon fiber conductive network still remains, as shown in Figure 3c,d. This unique porous mesoporous structure anchors polysulfide, which inhibits the “shuttle effect” and improves the cycling performance of the battery [32]. Figure 3e–h shows the EDS test analysis element map in the specified region of NPCNF/S, where C,N,S elements are evenly distributed, proving the uniformity of material doping.

**Figure 4:**
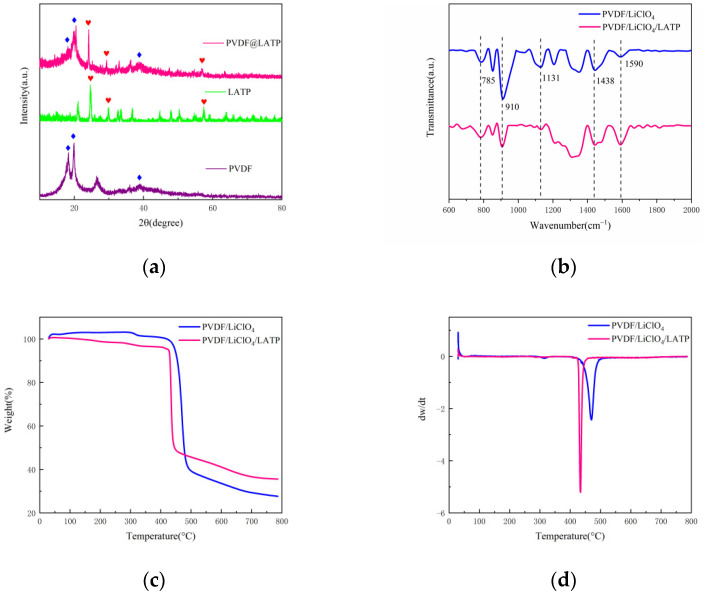
(**a**) XRD tests of the CPEs (**b**) ATR-FTIR (**c**) TGA curves of CPEs (**d**) DTG curves of CPEs.

The XRD results of CPEs can be seen in Figure 4a, 24.2° and 20°, respectively, correspond to characteristic peaks of LATP and PVDF, and another wide peak appears at 38.9°, indicating that PVDF is dominated by γ phase [33]. The characteristic peaks of PVDF and LATP were retained in the samples, indicating that the PVDF and LATP did not combine with each other, but kept their respective characteristics together. Figure 4b shows that the absorption peak of PVDF/LiClO_4_ complex at 785,910,1131,1438 and 1590 cm^−1^ did not shift with the addition of LATP. The change of peak value at 910 cm^−1^ corresponds to the out-of-plane bending of C-H bond, and the change of peak value at 1590 cm^−1^ is the stretching vibration of C-C bond and C=O, indicating that the addition of LATP is conducive to lithium-ion migration [34]. The TGA tests were conducted for PVDF/LiClO_4_ and PVDF/LiClO_4_/LATP. As shown in Figure 4c, rapid weight loss occurred at 400~500 °C. It can be seen from Figure 4d that the temperature of rapid sublimation loss of PVDF/LiClO_4_ and PVDF/LiClO_4_/LATP were 471 °C and 433 °C. The weight loss rate of PVDF/LiClO_4_/LATP was lower than that of PVDF/LiClO_4_, indicating that the addition of LATP improved the thermal stability of CPEs.

**Figure 5:**
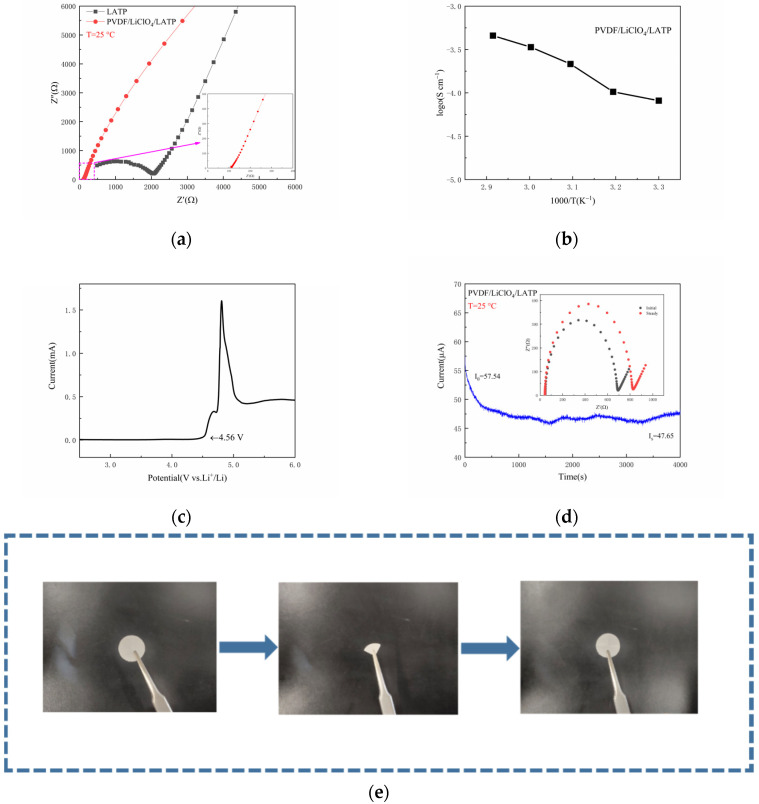
(**a**) EIS of CEPs; (**b**) Arrhenius plots of CEPs; (**c**) electrochemical window test; (**d**) time-ampere measurement symmetrical Li/CEPs/Li battery at 0.5 mV polarization and Nyquist plot initial/steady state internal illustration; (**e**) a photograph showing the flexibility of CEPs.

See Figure 5a for measuring the EIS of CPEs and LATP under 25 °C. It is obvious that the impedance of electrolyte was greatly reduced, which was due to the better flexibility of the membrane made by the combination of PVDF and LATP, as shown in Figure 5e, greatly reducing the interface impedance. The ionic conductivity was at 25 °C is 8.07 × 10^−5^ S cm^−1^. Figure 5b shows the Arrhenius diagram of CPEs. With the increase of temperature, the ionic conductivity also increased correspondingly. The increase of temperature promoted the expansion of the polymer and generated free volume in the polymer, which enhanced the segment movement of the polymer and increased the ionic conductivity [35]. The electrochemical window is also an indicator to evaluate the performance of CPEs. Therefore, linear sweep voltammetry (LSV) was used to characterize the electrochemical window. As shown in Figure 5c, the composite solid electrolyte membrane could withstand a voltage of 4.56 V, which is more than sufficient for Li-S batteries. Figure 5d shows the initial impedance spectrum and the impedance spectrum and timing current curve after polarization. The lithium-ion transfer rate of CPEs was calculated to be 0.77. Compared with a traditional liquid electrolyte (t_Li+_ < 0.5) [36], the addition of LATP improved the lithium-ion transfer rate and made the CPEs have better performance. A high lithium-ion transfer rate can generally reduce the concentration of movable anions in CPEs, thus reducing electrode polarization and the accompanying side reactions [37].

**Figure 6:**
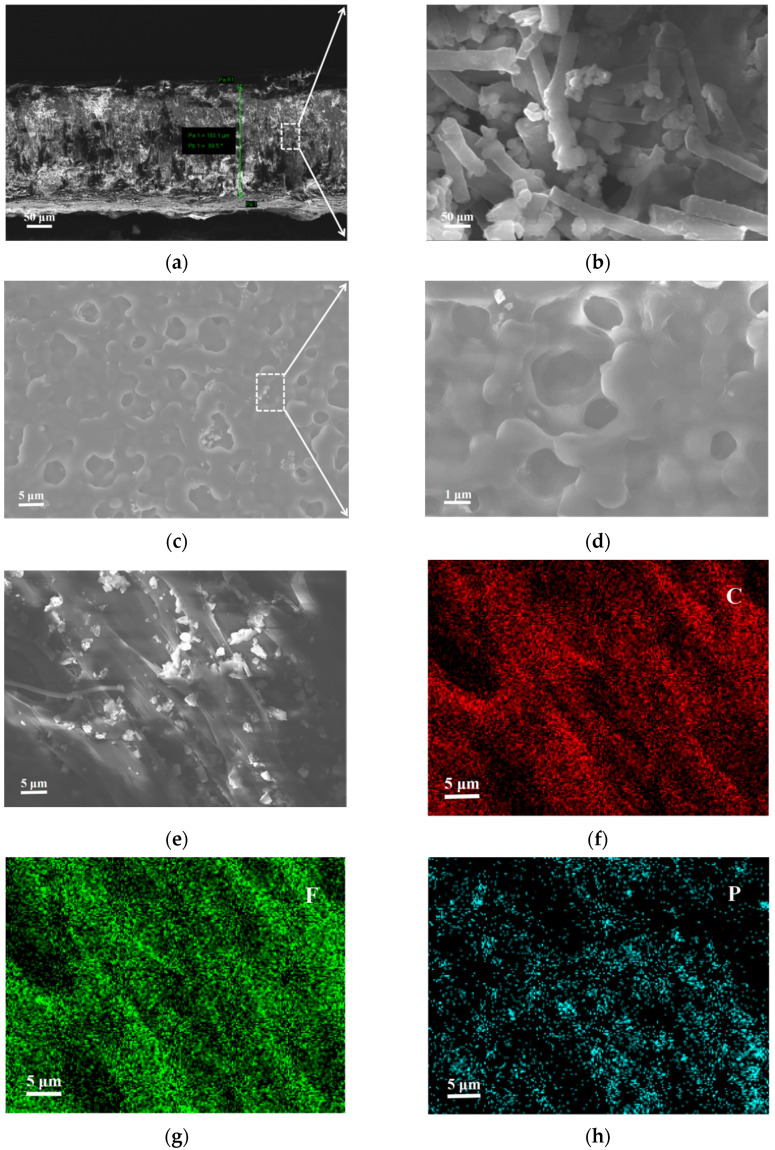
(**a**,**b**) A cross-sectional SEM image of CEPs; (**c**,**d**) SEM images of CEPs; (**e**) an SEM image of CEPs and corresponding elemental mapping images; (**f**) C element; (**g**) F element; (**h**) P element.

Figure 6 shows the CPEs interface and surface SEM characterization tests. Figure 6a,b shows the porous structure of CPEs, which is consistent with the SEM image of the surface in Figure 6c,d. The thickness of CPEs was 183.1 μm, and the porous structure formed by PVDF fiber winding nano-LATP particles was conducive to the transport of lithium-ions [38]. The EDS spectrum in Figure 6e–h shows the existence of element P, proving that LATP was uniformly distributed in CPEs, which itself was conducive to the formation of lithium-ion migration channels [39].

**Figure 7:**
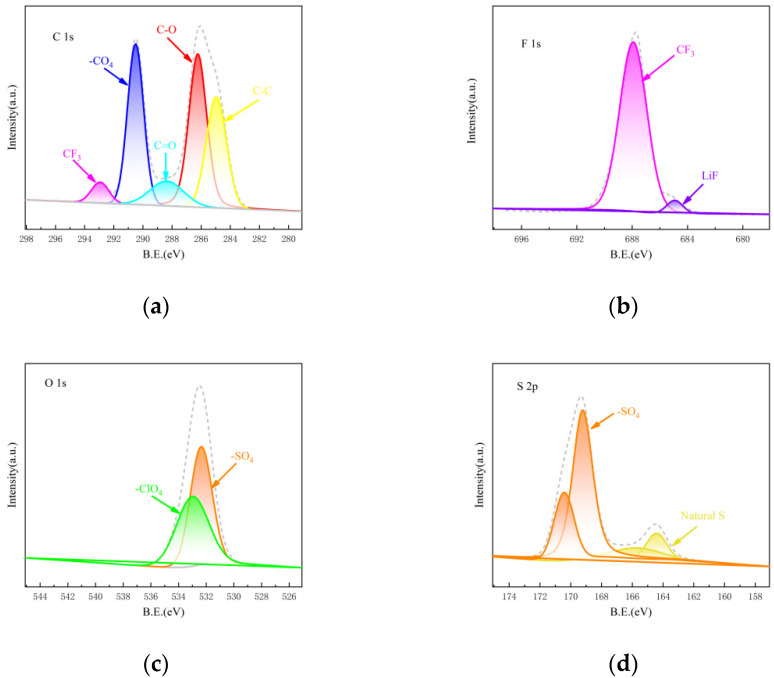
XPS of the CEPs after 500 cycles (**a**) C 1s (**b**) F 1s (**c**) O 1s (**d**) S 2p.

In order to further study the performance of CPEs, XPS tests were carried out on C, F, O and S elements in CPEs after 500 battery cycles. As shown in Figure 7a, the peak at 284.9 eV is the C-C bond peak of organic carbon, and the peak at 286.2, 288.3 and 290.5 eV are carbon–oxygen bonding peaks, indicating the existence of Li_2_CO_3_ in SEI film [40]. The characteristic peak of -CF_3_ appeared at 292.9 eV, corresponding to 684.9 eV in Figure 7b and indicating that -CF_2_ in PVDF underwent dehydrogenation to generate LiF [41], which exactly corresponded to the LiF peak at 687.9 eV in Figure 7b. The LiF can inhibit the growth of lithium dendrites and increase the diffusion rate of lithium-ions [42,43]. Figure 7c shows the O1s orbital graph. The characteristic peak of -ClO_4_ at 533 eV is the free -ClO_4_ in LiClO_4_. At 532.3 eV, -SO_4_ shows the positive S reaction to generate the sulfate salt, which is consistent with the -SO_4_ at 169.2 and 170.4 eV in Figure 7d. In Figure 7d, 164.4 and 165.6 eV are natural sulfur [44,45], and no peak bond of polysulfide is found, indicating that the PVDF/LiClO_4_/LATP electrolyte has a certain inhibitory effect on shuttle effect.

**Figure 8:**
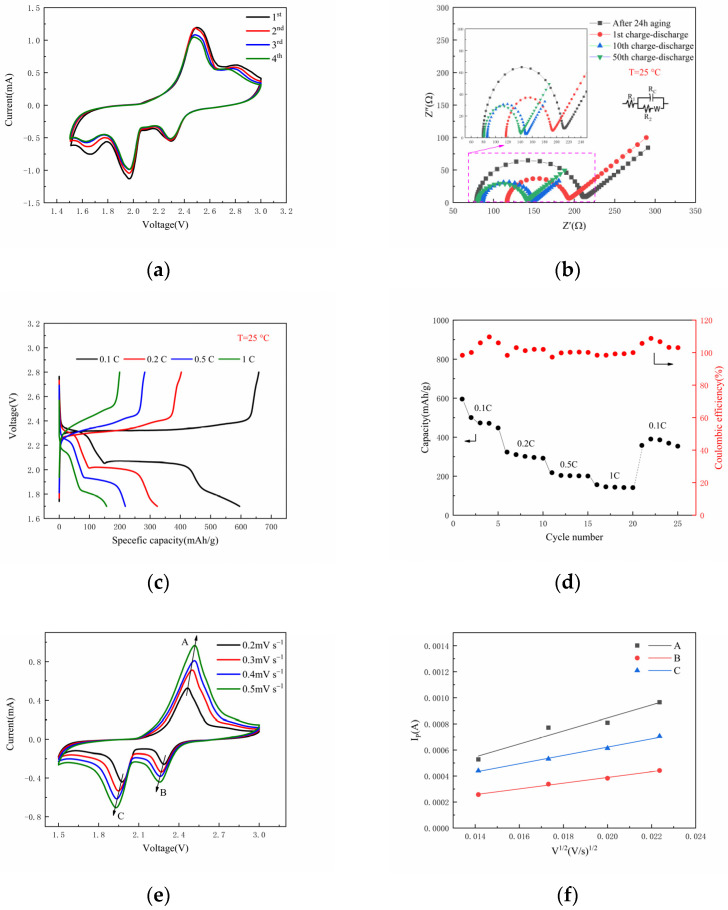

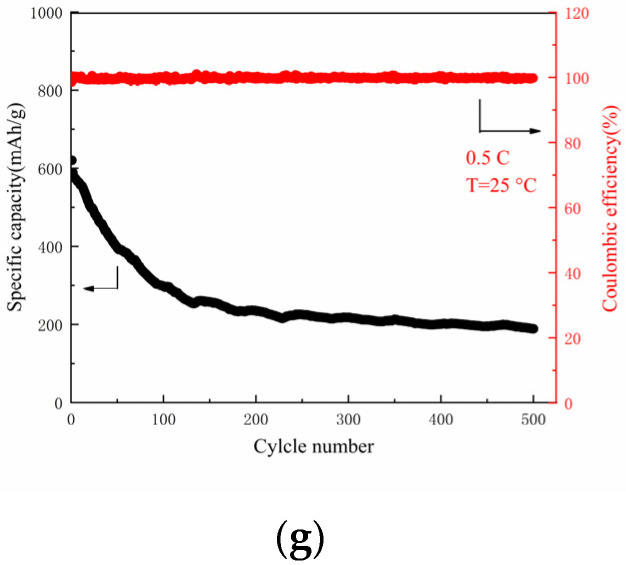
(**a**) CV curves of the Li|PVDF/LiClO_4_/LATP|NPCNF/S cell at a scan rate of 0.2 mV s^−1^; (**b**) EIS of the Li|PVDF/LiClO_4_/LATP|NPCNF/S cell at different cycle numbers; (**c**) voltage-specific capacity curve of the Li|PVDF/LiClO_4_/LATP|NPCNF/S cell; (**d**) rate performance; (**e**) CV curves of the cell in the range of 0.2–0.5 mV s^−1^; (**f**) corresponding linear fits of the peak currents of the cell; (**g**) cyclic performance of the Li|PVDF/LiClO_4_/LATP|NPCNF/S cell at 0.5 C.

Figure 8a presents the CV curves of the Li|PVDF/LiClO_4_/LATP|NPCNF/S batteries in the voltage range of 1.5~3 V at a scanning rate of 0.2 mV S^−1^ at 25 °C. In the first scan, two reduction peaks appear at 2.3 V and 1.98 V, indicating that S8 is reduced to Li_2_S_n_ (4 ≤ *n ≤* 8) and Li_2_S_2_/Li_2_S [46] during the discharge process. An oxidation peak that appeared at 2.5 V suggests Li_2_S_2_/Li_2_S oxidized in the process of charging. This is consistent with the phenomenon in the charge–discharge curve of Figure 8c. As the cycle continues, the SEI film tends to be stable and the test curves coincide well, which proves that the polymer electrolyte has good reversible properties. In order to further understand the electrochemical properties of solid-state lithium-sulfur batteries, the AC impedance after different charge–discharge cycles was measured, as shown in Figure 8b. All impedance spectra exhibit at least one semicircular with a Warburg component for the diffusion of lithium-ions through the electrode. After the first charge–discharge cycle, the RCT value was 193.5 Ω, as the number of cycles increased, Rct decreased and finally stabilized after the 10th cycle. It indicates that a stable SEI film is formed in the battery. Research regarding the rate performance test between 0.1 and 1 C is shown in Figure 8d. The discharge specific capacities of the Li|PVDF/LiClO_4_ LATP|NPCNF/S battery were 595.5 mAh g^−1^ (0.1 C, 1st), 292.3 mAh g^−1^ (0.2 C, 10st), 200.9 mAh g^−1^ (0.5 C, 15st), 141.5 mAh g^−1^ (1 C, 20st). When the rate was restored to 0.1 C, the specific capacity was 390.5 mAh g^−1^. This shows that the reversible specific capacity of the battery can be maintained after the charging and discharging cycle with a high rate, which proves that the battery has good rate performance. Moreover, the lithium ions diffusion coefficient can be obtained by a series of processing in terms of CV curves at different scan rates, shown in Figure 8e,f. The anodic and cathodic Li^+^ diffusion rate of D_(ALi+)_ = 1.06 × 10^−8^, D_(BLi+)_ = 2.06 × 10^−9^ and D_(CLi+)_ = 4.36 × 10^−9^ cm^2^ s^−1^. Figure 8g shows the test of 500 long cycles of the battery at 25 °C and 0.5 C. The capacity decay of only 0.139% per cycle, and the coulomb efficiency of the whole cycle is close to 100%, indicating that the battery has good cycle performance. The conductive framework in NPCNF/S and composite electrolytes (CPEs) can provide embedded channels for polysulfide formed in the charge–discharge cycle and inhibit the deposition of polysulfide at the Li-anode interface, thus reducing the influence of “shuttle effect” and improving the performance of batteries.

## 4. Conclusions

In this paper, NPCNF as a high-efficiency conductive skeleton of sulfur electrode active material was prepared by template method. The PVDF/LiClO_4_/LATP electrolyte with good performance was prepared via mixed solution casting method. The NPCNF has a good morphology, such as with nano fiber, having an obvious adsorption effect on polysulfide, and the sulfur content can reach 62.9 wt%. The active material has a high carbonation degree of graphite and good electrical conductivity. At 25 °C, the ionic conductivity of PVDF/LiClO_4_/LATP electrolyte is 8.07 × 10^−5^ S cm^−1^, and the lithium-ion transfer rate can reach 0.77. With the addition of nanoscale LATP, the overall performance of CPEs is better than that of a garnet type and PVDF-based solid electrolytes. The assembled cell has a low impedance, and the RCT value of the first ring is 193.5 Ω. The battery has a good rate performance and can work at 1 C, maintaing a certain specific capacity. At 25 °C and 0.5 C, the specific discharge capacity of 500 cycles is 620.52 mAh g^−1^, and the capacity decay rate of each cycle is only 0.139%. This method for preparing the excellent sulfur positive electrode, combined with the composite electrolyte membrane, provides a new idea for improving the long cycle performance of solid-state lithium-sulfur batteries at room temperature.

## Data Availability

The data that support the findings of this study are available upon reasonable request.
